# Static Magnetic Fields Regulate T-Type Calcium Ion Channels and Mediate Mesenchymal Stem Cells Proliferation

**DOI:** 10.3390/cells11152460

**Published:** 2022-08-08

**Authors:** Haokaifeng Wu, Chuang Li, Muqaddas Masood, Zhen Zhang, Esther González-Almela, Alvaro Castells-Garcia, Gaoyang Zou, Xiaoduo Xu, Luqin Wang, Guoqing Zhao, Shengyong Yu, Ping Zhu, Bo Wang, Dajiang Qin, Jing Liu

**Affiliations:** 1 CAS Key Laboratory of Regenerative Biology, Guangzhou Institute of Biomedicine and Health, Chinese Academy of Sciences, Guangzhou 510530, China; 2 Guangdong Provincial Key Laboratory of Stem Cell and Regenerative Medicine, Guangzhou Institute of Biomedicine and Health, Chinese Academy of Sciences, Guangzhou 510530, China; 3 School of Life Sciences, University of Science and Technology of China, Hefei 230026, China; 4 Guangdong Cardiovascular Institute, Guangdong Provincial People’s Hospital, Guangdong Academy of Medical Sciences, Guangzhou 510100, China; 5 Bioland Laboratory, Guangzhou 510005, China; 6 Key Laboratory of Biological Targeting Diagnosis, Therapy and Rehabilitation of Guangdong Higher Education Institutes, The Fifth Affiliated Hospital of Guangzhou Medical University, Guangzhou 510000, China

**Keywords:** static magnetic field, mesenchymal stem cell, T-type calcium ion channel, MAPK signaling pathway

## Abstract

The static magnetic fields (SMFs) impact on biological systems, induce a variety of biological responses, and have been applied to the clinical treatment of diseases. However, the underlying mechanisms remain largely unclear. In this report, by using human mesenchymal stem cells (MSCs) as a model, we investigated the biological effect of SMFs at a molecular and cellular level. We showed that SMF exposure promotes MSC proliferation and activates the expression of transcriptional factors such as *FOS* (Fos Proto-Oncogene, AP-1 Transcription Factor Subunit) and *EGR1* (Early Growth Response 1). In addition, the expression of signal-transduction proteins p-ERK1/2 and p-JNK oscillate periodically with SMF exposure time. Furthermore, we found that the inhibition of the T-type calcium ion channels negates the biological effects of SMFs on MSCs. Together, we revealed that the SMFs regulate T-type calcium ion channels and mediate MSC proliferation via the MAPK signaling pathways.

## 1. Introduction

In this era of economic and technological advancement, communities are clamoring for the development of physics-based medicine, which could provide novel treatment choices, particularly for patients with chronic diseases. Physical-related technologies have been developed for clinical applications, for example, electrotherapy or magnetic therapy, in the past 20 years. A static magnetic field (SMF) is a constant vector field that defines the magnetic impact on electrical currents and magnetizes materials on objects [1]. Indeed, an increasing number of clinically approved medical devices based on SMF, such as magnetically guided medical devices [2,3], miniature robots [4], and magnetic nanoparticles in microfluids and nanomechanics for drug delivery [2], have been developed and introduced into the medical and veterinary markets [5,6,7,8,9].

SMFs can trigger various biological effects. Previous studies showed that SMFs cause a variety of cellular effects such as cell orientation, proliferation, migration, and so on [10,11,12,13]. However, some biological effects of SMFs reported in the literature are not consistent or are even controversial [11,14]. Those diverse effects may be caused by differences in the magnetic field type, intensity, exposure time, and biological samples [15]. Recently, Biao Yu et al. demonstrated that SMFs could improve iron metabolism and prevents high-fat-diet-induced diabetes [16]. Interestingly, they showed that the different directions of SMFs have diverse effects on type 2 diabetes (T2D). These results further indicate the complexity and diversity of the biological effects of SMF. 

The potential role in treating disease by SMFs has also attracted the attention of scientists in the fields of stem cells and tissue engineering. Adult stem cells, key cells for tissue formation and organ regeneration, are central to tissue engineering and cell therapy [17,18]. Mesenchymal stem cells (MSCs) are adult stem cells that can be extracted from a variety of tissues, including bone marrow, adipose tissue, and tooth pulp [19]. They have received interest as a helpful tool for cell-based therapeutics due to their immunomodulatory capabilities and multilineage differentiation capability [20,21,22]. The effects of static magnetic fields on mesenchymal stem cells have been reported by many scientists [1,23,24]. It is intriguing to see whether SMF exposure impacts MSC fate as well as their underline mechanisms, not only in vitro but also in vivo, especially since the effects of SMFs on stem cell biology are still poorly understood. 

In this study, by using MSCs as a cell model, we investigated the biological effect of SMF and found that 72 h of exposure to an SMF promotes the mesenchymal stem cell proliferation rate significantly when compared to those without an external applied static magnetic field. Further mechanism study showed that the activation of the MAPK signaling pathway and the on/off switching events of the T-type calcium ion channel are involved in SMF-mediated MSC proliferation.

## 2. Materials and Methods

### 2.1. MSC Culture

The human umbilical cord-derived MSCs used in this project were isolated from Wharton’s Jelly, a gelatinous tissue around umbilical vessels. The human umbilical cord was obtained from the healthy cesarean section fetus. The MSCs samples were donated from The Fifth Affiliated Hospital of Guangzhou Medical University. MSCs were cultured in a medium with Dulbecco’s modified Eagle’s medium (DMEM)/F12 and 10% fetal bovine serum (FBS) in a 37 °C 5% CO_2_ incubator.

### 2.2. STORM Images Acquisition and Analysis

For imaging purposes, cells were plated in borosilicate glass-bottom 8-well chambers (Ibidi, Gräfelfing, Germany) at a confluency of 20,000–30,000 cells/cm^2^). Cells were fixed with PFA 4% (Alfa Aesar, Lancashire, UK) for 15 min at room temperature (RT) and then rinsed with PBS three times for 5 min each. Cells were permeabilized with 0.4% Triton X-100 (Acros Organics, Morris Plains, NJ, USA) in PBS for 15 min and rinsed with PBS three times for 5 min each. Cells were blocked in blocking buffer (10% BSA (Fisher Scientific, Branchburg, NJ, USA)–0.01% Triton X-100 in PBS) for 1 h at RT. Cells were incubated with primary antibody, mouse anti-H3 (Active Motif, Carlsbad, CA, USA), in incubation buffer (10% BSA–0.01% Triton X-100 in PBS) in a 1:50 dilution at 4 °C overnight. Cells were washed three times for 5 min each with washing buffer (2% BSA–0.01% Triton X-100 in PBS) and incubated for 1 h at RT with secondary antibody (Abcam, Cambridge, UK) in a 1:300 dilution in incubation buffer. Cells were washed three times for 5 min each with washing buffer before proceeding to imaging.

Imaging was performed on an NSTORM 4.0 microscope (Nikon, Tokyo, Japan) equipped with a CFI HP Apochromat TIRF 100 × 32 1.49 oil objective and an iXon Ultra 897 camera (Andor, Belfast, Northern Ireland) using highly inclined and laminated optical sheet illumination (HILO). Simultaneous imaging acquisition was performed (for every frame, a 647 nm reporter laser was used concurrently with a 405 nm laser in order to reactivate the reported dye) with a 10 ms exposure time for 60,000 frames. A 647 nm laser was used at a constant ~2 kW/cm^2^ power density. Single-color imaging was performed using a previously described imaging buffer [25], 100 mM Cysteamine MEA (Sigma-Aldrich, MA, USA), 5% glucose (Sigma-Aldrich, Burlington, MA, USA), 1% Glox (0.5 mg/mL glucose oxidase, 40 mg/mL catalase (Sigma-Aldrich, Burlington, MA, USA) in PBS. 

Localizations were extracted from raw images of STORM data using Insight3 standalone software (a kind gift from Bo Huang, UCSF). Cluster analysis was performed on the analyzed localizations as previously described [26].

### 2.3. RNA-Seq and Data Analysis 

Total RNA was isolated. The VAHTSTM mRNA-seq V3 Library Prep Kit for Illumina^®^ (NR611-01/02) was used to prepare sequencing libraries, and the libraries were sequenced on an Illumina Novaseq platform, and 150 bp paired-end reads were generated. 

Sequenced reads were aligned to the human Ensembl annotations transcriptome (v104) using Bowtie2 (version 2.4.4) and RSEM (version 2.4.1), then normalized using the TPM normalization method. Biological replicates were merged. Differentially expressed genes were obtained using a fold change of >1.1 as a threshold. Gene ontology analysis was performed using clusterProfiler (version 1.22.0).

### 2.4. Quantitative RT-PCR

TRIzol was used to extract the total RNA. HiScript II Reverse Transcriptase (Vazyme biotech R222-01) was used to reverse-transcribe cDNA from 2 μg of total RNA. The ChamQTM SYBR^®^ qPCR Master Mix (Vazyme biotech Q311-02, Nanjing, China) was used to perform real-time quantitative PCR. The values of relative expression were standardized to GAPDH. Technical triplicates were used for each experiment.

### 2.5. Western Blot Analysis

A total of 1 × 10^6^ MSCs were washed with PBS and trypsinized, collected in a 1.5 mL centrifuge tube, and washed again with PBS. The collected cells were resuspended in 100 μL lysis buffer containing 62.5 mM Tris-HCl, 10% glycerol, 2% SDS, 0.025% bromophenol blue, and 50 mM DTT with a protease-inhibitor cocktail (Roche) and incubated on ice for 30 min. After that, the mixture was centrifuged for 10 min, the supernatant was collected in another 1.5 mL of Appendrof, and the protein concentration was measured by using a NanoDrop 1000 spectrophotometer (Thermo Scientific, Waltham, MA, USA). Extracted cell protein samples were boiled for 10 min at 100 °C. The samples were separated by using 10–12% SDS-PAGE and transferred to a PVDF membrane (Amersham Biosciences, Piscataway, NJ, USA) using a wet transfer system. To probe, membranes were incubated with primary antibodies phospho ERK1/2 (Thr202/Tyr204) (Cell Signaling, Beverly, MA, USA), phospho JNK (Thr183/Tyr185) (Cell Signaling, Beverly, MA, USA), phospho P38 (Thr180/Tyr183) (Cell Signaling, Beverly, MA, USA); and GAPDH (Sigma-Aldrich, Bur-lington, MA, USA) at 4 °C for overnight, followed by appropriate HRP conjugated secondary antibodies and ECL detection. Western blotting results were obtained by the BioRad ChemiDoc TMXRS+ system and Beijing Tanon Fine-doX6. ImageJ software was used to quantify the protein relative level shown by Western blotting.

### 2.6. Flow Cytometry

The distribution of cells in different phases of the cell cycle after exposure to a magnetic field was analyzed with flow cytometry. Briefly, MSCs were harvested and washed with PBS after exposure for 0, 24, 48, 72, 96, 120, and 144 h to a magnetic field. The cells were fixed with 70% cold ethanol stored at −20 °C overnight and then stained with PI solution (consisting of 1 mg/mL PI and RNase A). The fluorescence-activated cells were sorted by flow cytometry (BD LSRFortessa), and the data were analyzed using CellQuest analysis software.

### 2.7. Measuring Resting Membrane Potential Using the Fluorescent Voltage Reporters DiBAC_4_(3)

DiBAC_4_(3) (bis-[1,3-dibutylbarbituric acid] trimethine oxonol; Invitrogen B438), excitation maxima of 490 nm and emission maxima of 516 nm. The anionic DiBAC_4_(3) exits the cell when the inner leaflet becomes more negative (hyperpolarized), and its signal declines. Conversely, when the inner leaflet becomes more positive, more DiBAC_4_(3) enters, increasing the intensity of the DiBAC_4_(3) signal (brighter) [27,28]. By analyzing the light intensity difference of the dye, we can determine whether the cell is experiencing depolarization or hyperpolarization. A 1 mM (1 mg/2 mL) stock solution was prepared in DMSO and stored in the dark at room temperature. For cell staining, a 5 μM final concentration was dropped in phenol-red-free media or buffer for 30 min before taking images. The dyes are very temperature sensitive; thus, the temperature was well controlled during the 30 min of incubation.

### 2.8. Statistical Information

Data are presented as mean ± s.d. as indicated in the figure legends. An unpaired two-tailed Student *t*-test was used, and the *p* value was calculated with Prism 6 software. A value of *p* < 0.05 was considered statistically significant; * *p* < 0.05, ** *p* < 0.01, and *** *p* < 0.001. No statistical method was used to predetermine the sample size. The experiments were not randomized. The investigators were not blinded to allocation during the experiment and outcome assessment.

## 3. Results

### 3.1. Static Magnetic Field Promotes the Proliferation of Mesenchymal Stem Cells

**Figure 1:**
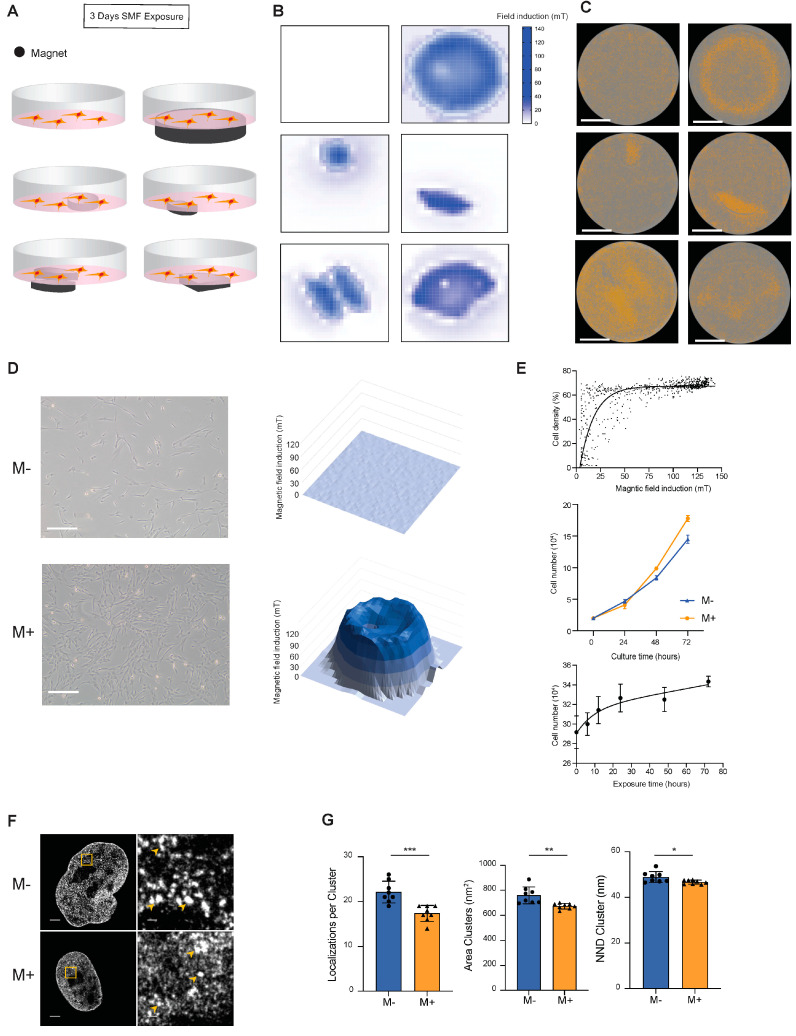
Exposure to SMF promotes MSC proliferation. (**A**) Schematic drawing of SMF exposure during MSC proliferation. (**B**) Heat maps showing the magnetic field strength of different shapes of permanent magnets. (**C**) Images of whole-well cell distributions influenced by SMF provided by different shapes of permanent magnets. Scale bars, 10 mm. (**D**) Morphology of MSCs under different magnetic field exposure conditions. M− indicates no external SMF; M+ indicates that a static magnetic field is applied. Scale bars, 100 μm. (**E**) Plots showing the relationship between the field strength, exposure time, culturing time, and cell numbers. Data are mean ± s.d., *N* = 3 independent experiments. (**F**) (Left) Cropped representative nuclear super-resolution images of MSCs labeled against H3 nucleosome under different magnetic field exposure conditions. Scale bars, 2000 nm. (Right) Magnification of the region inside the yellow box is shown. Yellow arrows indicate representative examples of nucleosome clutches. M− indicates no external SMF; M+ indicates that an SMF is applied. Scale bars, 200 nm. (**G**) Dot plots showing the median number of localizations per nucleosome clutch, median area per clutch, and the nearest neighbor distance (NND) between clutches for M−/M+ MSCs (*N* = 8). Asterisks indicate statistical significance of the separation between the mean of the medians according to unpaired two-tailed *t*-test between conditions. ns *p* > 0.05; * *p* < 0.05; ** *p* < 0.01; *** *p* < 0.001.

To investigate the biological effects of SMFs on MSCs, magnets of different shapes were used to create different magnetic environments in cell culture dishes (Figure 1A). In detail, MSCs were seeded at a density of 20,000 cells/well in six-well plates, then conventional permanent magnets NdFeB with different shapes were placed directly beneath the cell culture disks during the cell culturing. The precise SMF distribution was measured with a Gaussmeter by scanning the horizontal planes 1 mm (the thickness of the culture disk is 1 mm; hence, the cells and SMF were separated by 1 mm) above the magnets and then plotted by heatmaps (Figure 1B). The color bar in Figure 1B indicates the magnetic field induction with unit mT. After 72 h of culturing in an MSC medium, the cell distribution images were acquired and analyzed by the IncuCyte S3 Live-Cell Analysis System. The image results showed that when there is no external SMF during cell culturing, cells were distributed evenly over the well (Figure 1C). However, when different shapes of SMF were applied, cells accumulated densely on the tops of SMF regions (Figure 1C) and created patterns that reflect the geometry of the underlying SMF distribution. Those data indicated a clear cell-density difference between high-magnetic-gradient and low-magnetic-gradient regions. It was also reported by Zablotskii et al. that where cells were initially attracted to the regions with the highest magnetic gradient, then cells occupied the all top surface of the magnet [23]. Figure 1D (left) demonstrates the morphology of MSCs in the M−/+ group, and (right) demonstrates the magnetic field distribution of the M−/M+ group in a three-dimensional aspect.

We then analyzed the cell density distribution with respect to the magnetic field strength by merging the cell distribution image with corresponding SMF distribution heatmaps. The results show that there was a high correlation between the magnetic field induction and cell density (Figure 1E). The magnetic field strength is maximal at the magnet edges.

To investigate whether magnetic-field-dependent differences in cell distribution are caused by cell proliferation, we tested the cell proliferation rate by incubating MSCs continuously for 72 h under magnetic field exposure or not. In detail, in the M+ group, MSCs were seeded at a density of 200,000 cells/well in six-well plates (bottom area: 9.6 cm^2^) and circular NdFeB magnets with a maximum field intensity of 140 mT (surface area 7.1 cm^2^) were placed directly beneath the cell culture disks. In the control group (or M− group), no magnets were used. Samples were collected for cell counting on days 0, 1, 2, and 3. Together, the results clearly showed that the cell proliferation rate was approximately 23% higher in the M+ group than in the M− group under 72 h of culturing (Figure 1E). We further investigated the SMF exposure time effect on MSC proliferation by altering the exposure time. Samples were cultivated continuously for 72 h and separated into six groups according to various SMF exposure times: 0 h, 6 h, 12 h, 24 h, 48 h, and 72 h. As shown in Figure 1E, as the exposure time increases from 0 h to 72 h, the proliferation rate increases accordingly. This result indicates that increasing the exposure time acts as a cumulating effect to regulate the proliferation rate of MSCs.

We then investigated the effect of SMF on nuclear architecture. For that, we obtained super-resolution images of the chromatin structure. By using stochastic optical reconstruction microscopy (STORM), we can quantify small alterations in the chromatin structure, which have been previously linked to the cell state [26,29,30]. As shown in Figure 1F, the chromatin clusters, also known as clutches, were visualized by immunolabeling of the core histone H3. Chromatin clutches visually decrease their size and density after 72 h of 140 mT SMF exposure (Figure 1F, right). Dot plots showing the median number of localizations per nucleosome clutches, median area per cluster, and the nearest neighbor distance between clusters show a significative quantitative reduction in the number of molecules forming the clutches, the clutch size, and their distribution in the nucleoplasm (Figure 1G). These results are consistent with previous reports by Ricci et al., showing that rapidly proliferating stem cells have smaller chromatin clusters [26,29]. 

Together, these results suggest that the 140 mT SMF with 72h exposure time can increase the MSC proliferation rate by 23%.

### 3.2. Exposure to SMF Upregulates Immunoregulatory Factor Genes and MAPK-Signal-Pathway-Related Genes in Mesenchymal Stem Cells

**Figure 2:**
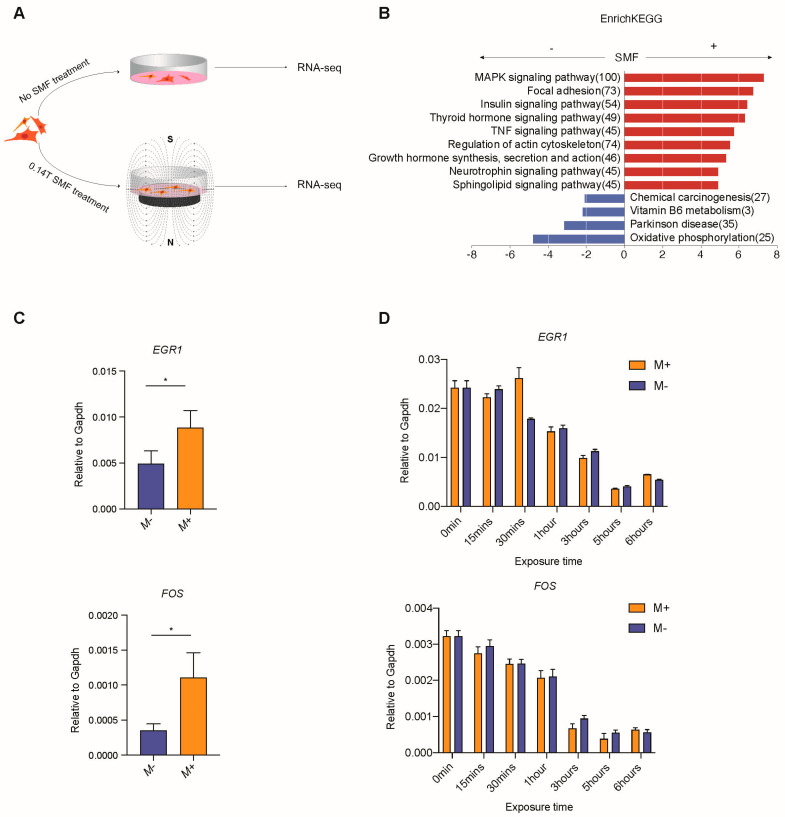
Exposure to SMF upregulates immunoregulatory factor genes and MAPK-signal-pathway-related genes in MSCs. (**A**) Schematic of experimental approach. (**B**) Bar chart of the gene expression differences between M+ and M− cell samples by RNA-seq of various KEGG function terms. (**C**) RT-qPCR analysis for expression of *EGR1* and *FOS* in M+ (magnetic field induction 140 mT) and M− MSCs samples cultured for 72 h. Asterisks indicate statistical significance of the separation between the mean of the medians according to unpaired two-tailed *t*-test between conditions. ns *p* > 0.05; * *p* < 0.05. (**D**) Time-course RT-qPCR analysis for expression of *EGR1* and *FOS* in M+ and M− MSCs samples cultured for 0–6 h. Data are mean ± s.d., *N* = 3 independent experiments.

To investigate the response of gene expression by SMF stimulation on MSCs, we performed RNA-seq for the samples of 72 h M+ or M− MSC cells (Figure 2A). The analysis results showed that the overall changes in gene expression are considered to be very mild; hence, the fold change was set to 1.1 (Figure 2B). The genes related to cell proliferation, such as *c-FOS* and *EGR1*, increased significantly when the MSCs were exposed to an SMF for 72 h. The GO analysis showed that the MAPK signaling pathway, focal adhesion, and growth hormone synthesis and secretion are enriched in M+ upregulated genes, while oxidative phosphorylation is enriched in M− upregulated genes (Figure 2B). The q-PCR analysis further confirmed the upregulation of *EGR1* and *FOS* by SMF stimulation (Figure 2C). Thus, we believe that 140 mT SMF induces the proliferation rate and, as a result, upregulates the expression of cell-growth-related genes such as *FOS*, *EGR1*, etc., via activation of the MAPK signaling pathway.

In addition, a 6-h time-course q-PCR for *FOS* and *EGR1* illustrated the changes in expression within the first 6 h after MSCs were seeded (Figure 2D). Both genes experienced a rapid drop in the first 5 h and then climbed up slightly at the 6th hour and remained consistent over 72 h. We did not observe a significant difference in *FOS* between M+ and M−, which means that SMFs do not play a crucial role in regulating *FOS* at the early stage. In contrast, M+ (magnetic field induction 140 mT) illustrated a notable upregulation in *EGR1* at a 30 min exposure time compared to M−.

### 3.3. SMF Exposure Time Modulates the Activation of MAPK Protein in Mesenchymal Stem Cells

**Figure 3:**
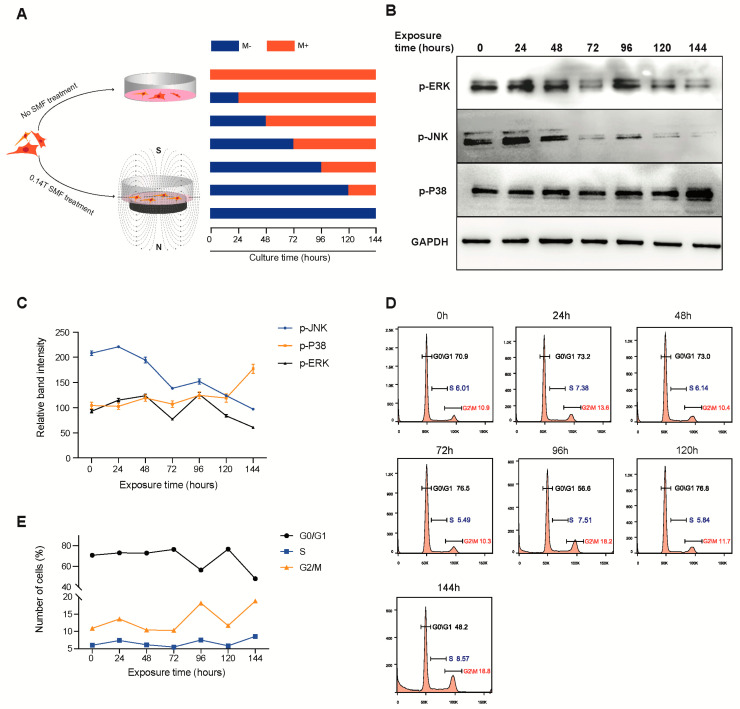
SMF exposure time modulates the activation of ERK and p-ERK protein in MSCs. (**A**) Schematic of experimental approach. (**B**) The representative results of Western blot of p-JNK, p-ERK, and p-P38 in MSCs cultured for 6 days, exposed at SMF for 0–144 h. (**C**) The graph showing relative band intensities of p-JNK, p-ERK, and p-P38 shown in (**A**). Data are mean ± s.d., *N* = 3 independent experiments. (**D**) The distributions of cell cycle after exposure for 0, 24, 48, 72, 96, 120, and 144 h to SMF were analyzed with flow cytometry. (**E**) The line chart of G0/G1, S, and G2/M phase distributions after exposure for 0, 24, 48, 72, 96, 120, and 144 h to 140 mT SMF.

The enrichment of the GO term MAPK signaling pathway for the M+ upregulated gene in Figure 2B raises the hypothesis that SMFs promote MSC proliferation by the MAPK signaling pathway. Since the MAPK signaling pathway is divided into three subclasses—ERK, P38, and JNK [31]—we detected their activation by Western blot. The experimental setup is illustrated in Figure 3A, and the results show that both p-ERK1/2 and p-JNK protein demonstrated a periodic oscillation according to the SMF exposure time. Both p-ERK1/2 and p-JNK experienced a continuous drop when exposed to SMF for 72 h, while a significant climb was observed when the SMF exposure time lasted for 96 h (Figure 3B,C) and oscillated back to a relatively lower expression level at 120 h and 144 h, whereas p-P38 dropped at 24 h of SMF exposure and climbed back to the original expression level when exposed for more than 24 h. Furthermore, cell cycles of samples with different exposure times were measured using flow cytometry (Figure 3D). The results for the cell-cycle distribution are resounding; as the SMF exposure time increases from 0 h to 144 h, the G2/M phase follows a similar trend to the p-ERK and p-JNK expression (Figure 3E). Such a transient change in p-ERK1/2 and p-JNK protein expression matches with the SMF-induced proliferation phenotype described in Figure 1.

These data indicate that the length of SMF exposure time triggers the MSC cell-cycle distribution via activation of the ERK/JNK-MAP kinase signaling pathway.

### 3.4. SMF Effecting Transmembrane Depolarization via T-Type Calcium Ion Channel

To discover the biosensors of the cell response to a magnetic field, we looked to the literature and found studies indicating that ion channels may represent one of the biosensors [32,33,34]. Here, we discovered a change in membrane potential (V_mem_) when the cells were exposed to 140 mT SMF for 30 min. Changes in the steady-states (resting) V_mem_ of non-excitable cells typically encode crucial instructive cues that regulate differentiation, proliferation, and cell-to-cell communication [35]. To this end, we applied a voltage-sensitive reporter dye DiBAC_4_(3), which only fluoresces inside the cell membrane and enters and exits the cell in reaction to the plasma membrane’s charge. The anionic DiBAC_4_(3) exits the cell when the inner leaflet becomes more negative (hyperpolarized), and its signal declines. Conversely, when the inner leaflet becomes more positive, more DiBAC_4_(3) enters, increasing the intensity of the DiBAC_4_(3) signal (brighter) [27,28]. The process of depolarization or hyperpolarization can be determined by analyzing the fluoresce intensity difference of the dye.

**Figure 4:**
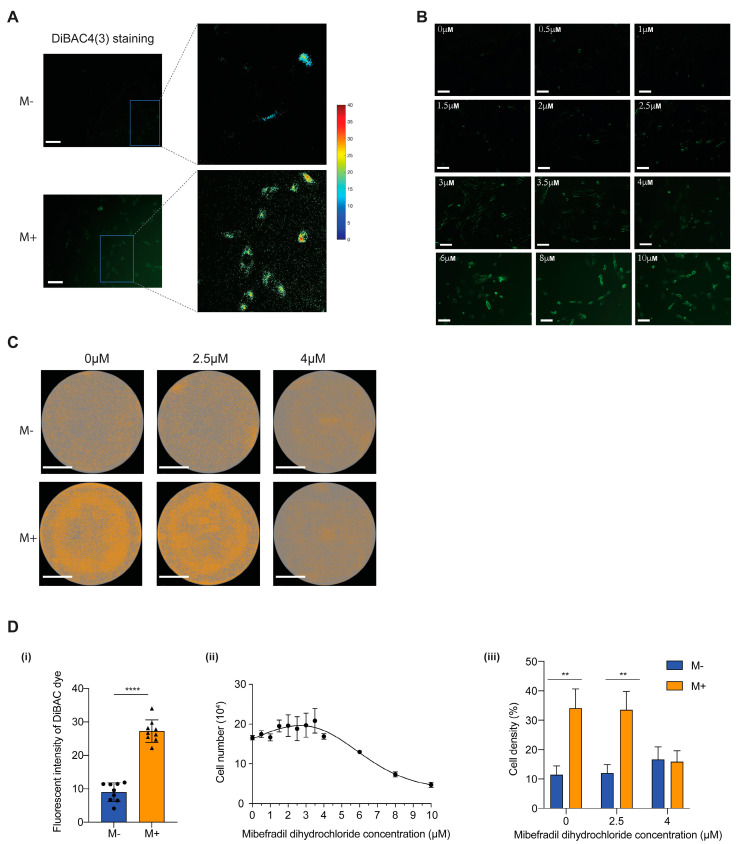
The SMF affects membrane depolarization via the T-type calcium ion channel. (**A**) The spatial distribution of V_mem_ across MSCs can be visualized by voltage-sensitive reporter dyes DiBAC_4_(3) to reveal depolarization when cells are exposed to SMF. The color bar indicates the fluorescent intensity of DiBAC_4_(3) dye. (**B**) The intensity of voltage-sensitive reporter dye DiBAC_4_(3) signal of MSCs treated with T-type Ca^2+^ channel blocker (CCB) mibefradil dihydrochloride. The fluorescence of the dye indicates the degree of depolarization. Scale bars, 100 μm. (**C**) Images of whole-well cell distributions when treated with different concentrations of CCB under an M+/M− environment. Scale bars, 10 mm. (**D**) Statistical analysis of Figure 4 A–C. (i) Bar chart indicating the intensity of voltage-sensitive reporter dye DiBAC_4_(3) signal difference between M+ and M−. (ii) Graph showing the relationship between cell number and mibefradil dihydrochloride concentration. (iii) Bar chart indicating the cell density of MSCs treated with different concentrations of CCB under an M+/M− environment. Asterisks indicate statistical significance of the separation between the mean of the medians according to unpaired two-tailed *t*-test between conditions. ns *p* > 0.05; ** *p* < 0.01; **** *p* < 0.0001.

As shown in Figure 4A (left), the spatial distribution of V_mem_ across cells can be visualized by voltage-sensitive reporter dye DiBAC_4_(3) to reveal the depolarization within the cell membrane. Figure 4A (right) shows a magnification of the region inside the blue box. The color bar in Figure 4A indicates the DiBAC_4_(3) fluoresces intensity. Here, Figure 4D(i) illustrates a stronger fluoresce signal in M+ than M−, representing that there are more positive ions flowing into the cell membrane, causing depolarization. In contrast, the DiBAC_4_(3) signal is much weaker when there is no SMF applied. This indicates that the SMF can significantly change the intracellular ion density, i.e., membrane potential, in a very short exposure time. Since V_mem_ is the sum of contributions from a variety of ion types, the same V_mem_ can be induced by a variety of channel proteins; the on/off switching events of various ion channels regulate the transmembrane flow of ions such as Na^+^, K^+^_,_ and Ca^2+^. Hence, it is worth investigating which ion channels respond to an SMF in our established system. 

The T-type calcium channel is a low-voltage-activated calcium channel that becomes inactivated during cell membrane hyperpolarization but then opens during depolarization [36]. In order to verify whether the T-type calcium ion channel is a crucial biosensor to SMF, we used a specific T-type Ca^2+^ channel blocker (CCB), mibefradil dihydrochloride, to inhibit the function of the T-type Ca^2+^ channel. The dosage was gradually increased from 0 μM to 10 μM and cultured for 3 days; then, the degree of depolarization was observed (Figure 4B). A clear increase in the fluorescence intensity was observed as the concentration increased, which indicates a greater degree of depolarization. The cell proliferation rate was recorded using Cell Counting Kit-8 per the manufacturer’s protocol. The results prove that a low concentration of CCB (~3.5 μM) increases the proliferation rate by about 25%, which has a similar effect on the proliferation rate as the 72-h SMF-treated MSCs. When the concentration was increased by more than 4 μM, the cell proliferation rate reduced dramatically, indicating the destruction of the T-type Ca^2+^ channel (Figure 4D(ii)) by mibefradil dihydrochloride due to over permeabilized membrane. Interestingly, when the CCB concentration reached 4 μM while exposed under SMF, there was no difference in the MSC proliferation rate between M+ and M− (Figure 4C,D(iii)). 

These results indicate that the T-type Ca^2+^ channel is an important membrane sensor for sensing magnetic fields.

## 4. Discussion

**Figure 5:**
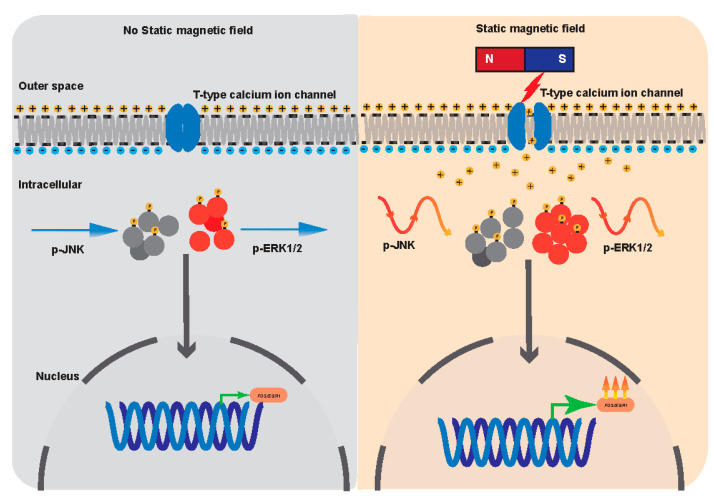
Graphic conclusion. Exposure to SMFs causes membrane depolarization transduced by T-type voltage-gated calcium channels into second-messenger cascades (e.g., ERK, JNK) that regulate downstream gene expression (e.g., *FOS*, *EGR1*, etc.); hence, directing the acceleration of the cell cycle promotes proliferation.

In this study, we investigated the biological effects of SMF on MSCs and found that SMFs promote the proliferation of MSCs. Specifically, MAPK signaling pathways are sensitive to SMF stimulation, and the activation of ERK and JNK signaling pathways seems to oscillate periodically with the exposure time of the SMF. Importantly, we demonstrated that T-type calcium ion channels mediated the SMF-dependent MSC proliferation (Figure 5). 

When an SMF is applied to cell systems, there are two types of magnetic forces that can act on subcellular components, molecules, and ions [32]: (1) Lorentz force. SMFs act forces on moving ions in the cells, causing electric field and currents to be induced. (2) Magnetic gradient force. When a non-uniform SMF is applied to the cells, this force is proportional to the gradient of the square of the magnetic induction. The cellular effects of SMFs are influenced by multiple parameters, which may consequently impact the experimental results. As a result, despite the fact that there are numerous in vitro and in vivo studies revealing the effects of magnetic fields on biological systems, there is still a lack of experimental coherence among them. However, the apparent inconsistencies are primarily due to the various magnetic field properties and experimental parameters, such as the magnetic field exposure time and intensity, among others. Varied forms of magnetic fields (static or dynamic, pulsed or noisy) as well as magnetic fields of different strengths (weak, moderate, or strong) or frequencies (very low frequency, low frequency, or radiofrequency) can all produce different and often completely opposing consequences [37,38,39]. In this study, the bioeffect of a single fixed SMF on MSCs was detected; the bioeffects of SMFs with different parameters need further study. 

Previously, it was reported that when rat cortical neuron cells were exposed to SMFs (ranging from 0.1 to 5 T), the ERK phosphorylation was upregulated when cells were exposed to 0.75 T but had no effect when exposed to 0.1 T, 0.5 T, 1 T, 2 T, and 5 T [40]. In addition, the ERK phosphorylation shows circadian oscillations in the mouse liver [41]. However, no reports demonstrated that different SMF exposure times modulate p-ERK and p-JNK periodic oscillation in human MSCs.

In our study, we found that MAPK signal transduction proteins p-ERK1/2 and p-JNK demonstrate a periodic oscillation under 0.14 T SMF according to their exposure time. The cell-cycle assay shows a similar oscillation pattern of the G2/M phase under the same magnetic field condition. The generality and cell specificity of this phenomenon need to be studied further. 

It has been shown that magnetic fields can affect the concentration of ions within the cytoplasm, including Ca^2+^ [42]. A study performed by Koch et al. demonstrated that extremely low-frequency (ELF) magnetic fields, ranging from 27 to 37 mT, can regulate Ca^2+^ transport by interacting with Ca^2+^ channels in the cell membrane [43]. Electromagnetic field (50 Hz, 20 mT) exposures led to the activation of Na^+^/K^+^ channels in MCSs, resulting in an increase in Na^+^/K^+^ concentration [44]. The modulation of key ion distributions consequently affects stem cell function, proliferation, and differentiation. The possible mechanisms of SMFs affecting membrane depolarization were mentioned in previous work by Zablotskii et al. [32]. In general, ion channels are gated by the Coulomb forces, which are much greater than the magnetic force. In a uniform SMF, the diffusion of charged ions (such as Na^+^, K^+^, Ca^2+^) can be affected by the Lorentz force and hypothetically change the membrane potential. However, an extremely high magnetic field (approximately 10^6^ T) [45] is required in order for the Lorentz force to suppress the Coulomb force. In a non-uniform SMF, the magnetic gradient forces induce an ion flux through the membrane that competes with those created by the gradients of electric potential and ion centration [33]. As a consequence, the magnetic gradient forces change the ion flux balance and membrane potential. According to the generalized form for the Nernst equation [33], the magnetic gradient field on the order of 10^9^ TM^−1^ can directly alter the membrane potential by 10 mV. In addition, the magnetic gradient field on the order of 10^3^ TM^−1^ can alter the activity of stretch-activated ion channels [46]; thus, the on/off switching events of ion channels can be regulated by the magnetic gradient forces. 

In summary, we showed that exposure to SMFs causes membrane depolarization transduced by T-type voltage-gated calcium channels into second-messenger cascades (e.g., ERK, JNK) that regulate downstream gene expression (e.g., *FOS*, *EGR1*, etc.); hence, directing the acceleration of the cell cycle promotes proliferation. Our findings reveal new cellular biological effects and signal transduction mechanisms of SMF. 

## Data Availability

The data used to support the findings of this study are included with the article.
